# Emerging Processing, Preservation, and Quality-Monitoring Technologies: Toward Sustainable and Intelligent Agri-Food Systems

**DOI:** 10.3390/foods15172961

**Published:** 2026-08-23

**Authors:** Liuyang Shen, Xianzhe Zheng, Bing Lu, Dianbin Su

**Affiliations:** 1 College of Mechanical and Electronical Engineering, Tarim University, Alar 843300, China; feiyanghero@163.com; 2 College of Agricultural Equipment and Energy Engineering, Northeast Agricultural University, Harbin 150030, China; 3 College of Engineering and Technology, Southwest University, Chongqing 400715, China; lb20240908@swu.edu.cn; 4 School of Agricultural Engineering and Food Science, Shandong University of Technology, Zibo 255000, China; sdb@sdut.edu.cn

## 1. Introduction

The global agri-food system is navigating an era of unprecedented complexity and urgency. Rising population, accelerating climate change, depleting natural resources, and shifting consumer expectations for safer, more nutritious, and sustainably produced foods have collectively increased pressure on every link of the food supply chain [1,2,3]. These interconnected challenges require a fundamental rethinking of how agri-food products are processed, preserved, monitored, and optimized, moving beyond incremental improvements toward transformative, systems-level innovation [4,5].

In response to this imperative, the food science and engineering community has accelerated the development of a diverse range of advanced technologies across the agri-food continuum [6,7]. These include emerging processing technologies such as microwave, radio frequency, ultra-high pressure, three-dimensional (3D) food printing, cold plasma, and ultrasound-assisted techniques for pesticide degradation and microbial inactivation [8,9]; novel preservation strategies such as edible coatings and biodegradable packaging solutions to extend the shelf life of perishable foods [10,11]; intelligent quality-monitoring systems using optical detection, biosensors, and IoT-enabled sensors to evaluate freshness, maturity, physicochemical properties, and real-time food safety [12,13,14]; and computational modeling approaches including computer simulation, AI-driven numerical modeling, and machine learning to predict and optimize food processing performance [15,16]. Collectively, these innovations have the potential to improve efficiency, reduce waste, improve product quality, and strengthen food security, which are cornerstones of a resilient and sustainable food future [17,18,19].

Despite the remarkable progress achieved in recent years, persistent knowledge gaps continue to impede the translation of laboratory-scale breakthroughs into industrial practice [20]. The scalability of emerging processing technologies remains inadequately addressed; the integration of multi-source data for comprehensive, real-time quality evaluation is still in its infancy [21,22]; and the full potential of AI-driven predictive modeling to account for the multifactorial nature of food matrices, including biochemical, physical, and microbial dynamics, has yet to be realized [23,24,25]. There is an urgent requirement for preservation approaches that are sustainable and capable of prolonging shelf life without diminishing nutritional quality or sensory acceptability, alongside integrated frameworks that can bring these separate technological strands together into coherent, data-informed manufacturing systems [26,27].

Against this backdrop, we launched this Special Issue, “Advanced Technologies and Applications for Processing, Preservation, Quality Monitoring, and Computational Modeling of Agri-Food Products.” Our overarching objective was to bridge the persistent gap between theoretical research and practical industrial application while fostering interdisciplinary collaboration among food scientists, engineers, and data analysts. We sought to curate a collection of high-quality original research that showcases state-of-the-art innovations and their tangible contributions to optimizing processing parameters, extending shelf life, enabling real-time quality monitoring, and simulating complex food matrices. By bringing together innovations across these domains, we aimed to advance sustainable practices, improve food safety, and support the development of smart, data-driven solutions for the agri-food industry.

The five contributions ultimately assembled in this Special Issue, spanning intelligent quality-monitoring and 3D-food-printing technologies, emerging non-thermal processing technologies, and innovative preservation and shelf-life extension strategies, collectively embody the thematic breadth and depth envisioned in our original call. Pedreschi et al. [28] showed how computer vision can serve as a rapid, non-destructive tool for predicting neo-formed contaminants in baked crackers while also linking processing parameters with consumer preferences. Wang et al. [29] advanced non-destructive fruit quality evaluation by integrating near-infrared spectroscopy with explainable machine learning for multi-parameter prediction. In the study by Zhang et al. [30] on peanut butter, natural biopolymers were used to improve product stability and shelf life through formulation innovation and kinetic modeling. Zhang et al. [31] investigated the application of pulsed electric field technology in traditional meat processing and found that it can improve texture and flavor beyond what conventional thermal treatments can achieve. Liu et al. [32] extended personalized food manufacturing through the 3D food printing of cereal–legume starch-based gels, identifying optimal formulations for printing fidelity and sensory acceptance. Collectively, these studies engage directly with the core thematic pillars of this Special Issue while also indicating the wider direction of agri-food research, which is progressively bringing together real-time sensing, data-driven modeling, innovative processing technologies, and consumer-centered optimization within integrated intelligent manufacturing systems. In this manner, they highlight the indispensable importance of cross-disciplinary cooperation in confronting the principal problems facing the worldwide food system while also pointing to concrete pathways toward a food future that is more intelligent, safer, and increasingly sustainable [27,33].

Within this context, this Special Issue was initiated with two objectives: to bring together high-caliber original studies and to demonstrate how state-of-the-art innovations may be converted into practical solutions throughout the full agri-food chain. The five papers assembled in this collection—covering intelligent detection, innovative processing, advanced preservation, and computational methods—jointly reflect this intention and present a varied outlook on the future direction of food science and technology. Taken together, these studies align with the specific themes set out in our call and further emphasize the rising need for interdisciplinary integration in addressing the major challenges of global food security. In the following sections, each contribution is briefly summarized, and readers are invited to review the full texts to better understand the scope and significance of the advances presented.

## 2. An Overview of the Published Articles

The present Special Issue brings together five papers that tackle different but closely related problems across the wider domain of advanced agri-food technologies. Collectively, these contributions can be grouped into three main themes: (i) intelligent quality monitoring and computational modeling; (ii) innovative preservation and shelf-life extension strategies; and (iii) emerging non-thermal processing and 3D-food-printing technologies. The following paragraphs briefly introduce each of these contributions, aiming to spark the curiosity of readers to explore the full texts.

### 2.1. Intelligent Quality Monitoring and Computational Modeling

Accurate, rapid, and non-destructive quality assessment remains a cornerstone of modern food production. In this context, Pedreschi et al. [28] (Contribution 1) address a critical food safety concern—the formation of neo-formed contaminants (NFCs) during thermal processing. Crackers, a widely consumed snack, are susceptible to acrylamide (AA) and 5-hydroxymethylfurfural (HMF) formation through non-enzymatic browning reactions during baking. Conventional analytical methods for quantifying these contaminants are complex, labor-intensive, and require specialized personnel. To overcome these limitations, the authors developed computer vision (CV) models based on surface digital image analysis for the rapid prediction of AA and HMF in crackers. Across five baking temperatures (160–200 °C) and five baking times (15–35 min), CV estimates were compared with conventional analytical measurements using cross-validation with a “leave-one-treatment-out” approach. The average error for missing measurements was remarkably low, at 3.10% for AA and 3.28% for HMF, validating CV as an efficient tool for rapid NFC estimation. Importantly, consumer preference testing using the Check-All-That-Apply (CATA) method revealed that samples baked at 180 °C for 25 min—which also exhibited the lowest levels of both AA and HMF—were the most preferred. This work demonstrates the powerful synergy between rapid optical sensing and consumer science in optimizing food processing parameters.

Complementing this, Wang et al. [29] (Contribution 2) addressed the challenge of non-destructive quality evaluation in fresh fruit, focusing on ‘Dinosaur Egg’ Apricot plum. Using near-infrared spectroscopy (NIRS) with cross-parameter feature fusion, the study developed predictive models for soluble solids content (SSC), moisture content (MC), and fruit firmness (FF)—three key quality attributes determining market value and consumer acceptance. Spectral data were preprocessed, and key bands were screened via Competitive Adaptive Reweighted Sampling (CARS) and the Shuffled Frog Leaping Algorithm (SFLA). Partial Least Squares Regression (PLSR) models were established, and chemical index features were fused with FF-related preliminary features, with SHapley Additive exPlanations (SHAP) being employed to optimize feature contribution. The final models demonstrated high predictive performance: SSC ($R_{c}^{2}$ = 0.9354, $R_{p}^{2}$ = 0.9302), MC ($R_{c}^{2}$ = 0.9367, $R_{p}^{2}$ = 0.9314), and FF ($R_{c}^{2}$ = 0.8151, $R_{p}^{2}$ = 0.7986). This strategy significantly improves multi-quality detection accuracy, particularly for firmness, and provides robust technical support for intelligent fruit grading systems.

### 2.2. Innovative Preservation and Shelf-Life Extension Strategies

Extending the shelf life of perishable and semi-perishable foods while maintaining quality is a persistent challenge. Zhang et al. [30] (Contribution 3) addressed a persistent challenge in the plant-based food sector—oil separation in peanut butter, which accelerates oxidative rancidity and reduces shelf life. The study investigated the effect of soluble soybean polysaccharides (SSPSs) on the quality and shelf life of peanut butter. Optimal processing conditions were established by adding 1.7% SSPSs (*w*/*w*), heating to 85 °C for 40 min, and cooling to 1 °C. SSPS incorporation significantly increased product lightness without altering red–green color characteristics while also improving textural properties through increased hardness and cohesiveness. Nutritional analysis revealed elevated proximate composition parameters (moisture, ash, carbohydrates, and fiber), alongside slightly reduced acid and peroxide values. Scanning electron microscopy demonstrated that SSPSs enhanced the internal network structure, inhibited oil migration, and reduced centrifugal emulsification rates. First-order kinetic models based on acid and peroxide values were developed to predict shelf-life extension effects, with both model predictions and experimental data confirming that SSPS addition effectively prolongs peanut butter shelf life. This study highlights the coupling of food formulation innovation with kinetic modeling for predicting shelf life. It further demonstrates that natural biopolymers may function as efficient stabilizing agents, thereby responding to consumer interest in clean-label preservation approaches.

### 2.3. Emerging Non-Thermal Processing and 3D-Food-Printing Technologies

The pursuit of energy-efficient processing methods that preserve nutritional and sensory quality has catalyzed interest in non-thermal technologies. Zhang et al. [31] (Contribution 4) explored the application of pulsed electric field (PEF), a novel non-thermal processing technology, in meat processing. The study examined the effects of PEF pretreatment at varying electric field strengths (1, 2, and 3 kV/cm) and durations (30, 60, and 90 s) on the color, texture, moisture distribution, free amino acids, and flavor compounds in air-dried duck meat. PEF pretreatment significantly increased brightness (*p* < 0.05), while the optimal treatment (3 kV/cm, 30 s) improved textural properties by reducing chewiness and hardness by 65.44% and 59.97%, respectively. Mechanistic analysis revealed that PEF promoted myofibril disruption and vacuolization, reducing water mobility and improving moisture retention. Enhanced endogenous enzyme activity under PEF facilitated protein degradation, boosting total free amino acid content—particularly umami and sweet amino acids (e.g., glutamic acid and alanine). PEF pretreatment also elevated key aroma compounds, including hexanal, methyl caprate, and 4-methyl valerate, thereby improving the overall flavor profile of air-dried duck meat. This study provides comprehensive technical support for integrating PEF technology into traditional meat processing to achieve superior quality outcomes.

In parallel, the study on 3D food printing by Liu et al. [32] (Contribution 5) evaluated the 3D printability and rheological behavior of cereal–legume starch-based gels formulated with germinated brown rice flour (GBRF) and red adzuki bean flour (RABF), supplemented with xanthan and guar gum as functional additives. Comprehensive characterization was performed through Fourier transform infrared spectroscopy (FT-IR), rheology, texture analysis, scanning electron microscope (SEM), and sensory evaluation. The results demonstrated pseudoplastic behavior across all gel formulations, with the RABF/GBRF ratio of 1:2 (RG1:2) showing optimal color properties (ΔE* = 0.60 ± 0.86) and the 2:1 ratio (RG2:1) exhibiting superior printing fidelity and structural stability, achieving a printing accuracy of 99.37 ± 0.39%. Mechanical properties such as hardness and chewiness were significantly influenced by RABF/GBRF ratios, with RG2:1 exhibiting the highest hardness (1066.74 ± 102.09 g) and RG1:2 showing the best springiness (0.64 ± 0.10). Sensory evaluation indicated that the RABF/GBRF ratios of 1:1 and 1:2 received relatively high overall acceptance scores. These findings demonstrate that specific RABF/GBRF ratios can significantly improve 3D printability and textural properties, providing valuable insights for developing personalized and functional cereal–legume starch-based foods using 3D-food-printing technology.

## 3. Future Perspectives

Taken together, the contributions to this Special Issue point to several promising directions for future research that merit attention from the agri-food research community.

Integrating rapid sensing tools with consumer-oriented optimization represents one of the most promising directions. Pedreschi et al. [28] demonstrated that computer vision may serve not only as a quality-control instrument but also as a means of connecting processing variables with consumer preferences. In future studies, real-time optical sensing should be linked with adaptive process control so that product quality and safety can be optimized dynamically.

Further progress is warranted in multi-parameter quality prediction using spectroscopic methods and machine learning, as illustrated by Wang et al. [29]. By combining predictive modeling with SHAP-based interpretability, a more transparent and trustworthy AI framework for food quality assessment can be established. Future investigations should prioritize the generalizability of these models across fruit cultivars and cultivation conditions.

Developing clean-label ingredients to enhance food stability and extend shelf life, as Zhang et al. [30] showed through the use of SSPSs in peanut butter, aligns with rising consumer preference for minimally processed products containing familiar ingredients. Future work should examine whether comparable polysaccharide-based approaches can be applied to a wider range of food matrices, with particular attention to the mechanistic basis of structure–function relationships.

The incorporation of emerging non-thermal methods, including PEF, into conventional meat processing, as examined by Zhang et al. [31], creates additional avenues for quality enhancement that extend beyond standard heat-based treatments. Subsequent investigations ought to evaluate the technology’s scalability, its cost-effectiveness, and the extent to which it can be paired with other processing approaches to produce synergistic outcomes.

Progress in 3D food printing for personalized nutrition, as developed by Liu et al. [32], marks a promising and rapidly evolving area. Future work should focus on producing printable inks with better nutritional characteristics, enabling multi-material printing, and confirming the performance of 3D-printed foods in both clinical and community environments. In food systems, AI should likewise move past prediction and toward autonomous decision making, while large language models and generative AI are applied to recipe refinement and individualized nutrition.

The integration of these diverse technologies into cohesive, data-driven manufacturing systems remains a major challenge. Bringing together optical sensing, machine learning, novel processing technologies, and advanced formulation science will be essential to realizing the vision of smart, sustainable, and personalized food production.

## 4. Conclusions

The contributions to this Special Issue collectively show the transformative potential of advanced technologies across multiple domains of agri-food science. From rapid, AI-enhanced detection systems to precision non-thermal processing and natural preservation approaches, these studies address critical knowledge gaps and offer practical pathways for industry adoption. In particular, the integration of computer vision and explainable machine learning into quality-monitoring frameworks addresses the long-standing need for real-time, interpretable assessment tools. The application of PEF and 3D food printing shows how physical and additive manufacturing innovations can redefine product quality and personalization. Meanwhile, the use of natural biopolymers for shelf-life extension responds to growing consumer and regulatory demand for clean-label, sustainable solutions. We hope that the insights presented herein will inspire further interdisciplinary collaboration among food scientists, engineers, and data analysts, ultimately contributing to the development of smarter, safer, and more sustainable agri-food systems.

## Data Availability

No new data were created or analyzed in this study. Data sharing is not applicable to this article.
